# Estimation of tulathromycin depletion in plasma and milk after subcutaneous injection in lactating goats using a nonlinear mixed-effects pharmacokinetic modeling approach

**DOI:** 10.1186/s12917-016-0884-4

**Published:** 2016-11-18

**Authors:** Zhoumeng Lin, Matthew Cuneo, Joan D. Rowe, Mengjie Li, Lisa A. Tell, Shayna Allison, Jan Carlson, Jim E. Riviere, Ronette Gehring

**Affiliations:** 1 Institute of Computational Comparative Medicine (ICCM), Department of Anatomy and Physiology, College of Veterinary Medicine, Kansas State University, 1800 Denison Avenue, P200 Mosier Hall, Manhattan, KS 66506-5802 USA; 2 Department of Population, Health and Reproduction, College of Agricultural and Environmental Sciences, University of California, Davis, CA 95616 USA; 3 Department of Medicine and Epidemiology, College of Agricultural and Environmental Sciences, University of California, Davis, CA 95616 USA; 4 School of Veterinary Medicine, and Department of Animal Science, College of Agricultural and Environmental Sciences, University of California, Davis, CA 95616 USA; 5 Present address: Department of Pharmaceutical Science, College of Pharmacy, University of Oklahoma Health Sciences Center, Oklahoma City, OK 73117 USA

**Keywords:** Tulathromycin, Withdrawal time, Goat, Food safety, Nonlinear mixed-effects (NLME) pharmacokinetic modeling

## Abstract

**Background:**

Extra-label use of tulathromycin in lactating goats is common and may cause violative residues in milk. The objective of this study was to develop a nonlinear mixed-effects pharmacokinetic (NLME-PK) model to estimate tulathromycin depletion in plasma and milk of lactating goats. Eight lactating goats received two subcutaneous injections of 2.5 mg/kg tulathromycin 7 days apart; blood and milk samples were analyzed for concentrations of tulathromycin and the common fragment of tulathromycin (i.e., the marker residue CP-60,300), respectively, using liquid chromatography mass spectrometry. Based on these new data and related literature data, a NLME-PK compartmental model with first-order absorption and elimination was used to model plasma concentrations and cumulative excreted amount in milk. Monte Carlo simulations with 100 replicates were performed to predict the time when the upper limit of the 95% confidence interval of milk concentrations was below the tolerance.

**Results:**

All animals were healthy throughout the study with normal appetite and milk production levels, and with mild-moderate injection-site reactions that diminished by the end of the study. The measured data showed that milk concentrations of the marker residue of tulathromycin were below the limit of detection (LOD = 1.8 ng/ml) 39 days after the second injection. A 2-compartment model with milk as an excretory compartment best described tulathromycin plasma and CP-60,300 milk pharmacokinetic data. The model-predicted data correlated with the measured data very well. The NLME-PK model estimated that tulathromycin plasma concentrations were below LOD (1.2 ng/ml) 43 days after a single injection, and 62 days after the second injection with a 95% confidence. These estimated times are much longer than the current meat withdrawal time recommendation of 18 days for tulathromycin in non-lactating cattle.

**Conclusions:**

The results suggest that twice subcutaneous injections of 2.5 mg/kg tulathromycin are a clinically safe extra-label alternative approach for treating pulmonary infections in lactating goats, but a prolonged withdrawal time of at least 39 days after the second injection should be considered to prevent violative residues in milk and any dairy goat being used for meat should have an extended meat withdrawal time.

**Electronic supplementary material:**

The online version of this article (doi:10.1186/s12917-016-0884-4) contains supplementary material, which is available to authorized users.

## Background

Drug residues in edible tissues of food-producing animals are a global food safety concern [1, 2]. Tulathromycin is a widely used long-acting triamilide antibiotic labeled for treating respiratory diseases in cattle and swine, and also commonly used in goats in an extra-label manner because it is highly effective against caprine respiratory pathogens [3] and is clinically safe when administered to neonatal dairy and meat goats at 1X, 3X, and 5X the cattle label dose [4]. In the US and Europe, there is no established milk tolerance (equivalent to maximum residue level in Europe) for tulathromycin in goats, thus any detectable residues are considered violative. Therefore, it is important to determine the appropriate withdrawal time needed for tulathromycin concentrations in edible tissues/milk to fall below legally established safe levels (in this case the analytical detection limit) to ensure food safety.

Physiologically based pharmacokinetic (PBPK) and nonlinear mixed-effects pharmacokinetic (NLME-PK) models are helpful tools in the establishment of withdrawal time guidelines for veterinary drugs [5–7]. Currently, there are pharmacokinetic data for tulathromycin in the plasma and tissues of juvenile and market-age goats after single or multiple intravenous (IV), intramuscular (IM), or subcutaneous (SC) injections [3, 8, 9]. A PBPK model has been developed to help estimate meat withdrawal times for tulathromycin in juvenile and market-age goats [10]. For lactating goats, however, there are only pharmacokinetic data in the plasma and milk after single IV, IM, or SC injection [11, 12], but not after repeated administrations. PBPK or NLME-PK models for tulathromycin in lactating goats are not available, either.

Repeated administrations are a common and important dosing strategy in order to maintain therapeutically effective drug concentrations in the plasma and tissues for longer periods of time. A recent study suggested that in order to maintain lung concentrations of tulathromycin higher than minimal inhibitory concentrations (MIC) for respiratory pathogens (e.g., MIC = 2 μg/mL for *M haemolytica*), the SC injection interval in goats should be every 7 days [9]. A major concern of repeated administrations of veterinary drugs in food-producing animals is the potential accumulation of drugs in the body, resulting in prolonged withdrawal times. The objective of this study was to determine the pharmacokinetic characteristics of tulathromycin in lactating goats after two administrations, and to develop a NLME-PK model to estimate tulathromycin depletion in plasma and milk samples after single and two SC injections in lactating goats.

## Methods

### Pharmacokinetic study design

Eight healthy adult (1–7 years old) lactating goats with a mean body weight of 88 kg (range: 73–109 kg) and a mean milk production of 10.8 lbs/day (range: 7–14 lbs/day) in their first to sixth lactation were used in the present study. Breeds included Saanen (*n* = 4), LaMancha (*n* = 3), and Toggenburg (*n* = 1) goats. Does were housed individually in the Goat Teaching and Research Facility at University of California, Davis, CA. All does met the inclusion criteria, including passing a normal physical examination, lactating for at least 45 days, and no antibiotic treatment in the last 2 months. Prior to tulathromycin injection, composite milk samples representing one entire milking period (instead of foremilk or strippings) were collected using a Dairy Herd Improvement Association-approved milk metering device. Goats were then injected with 2.5 mg/kg tulathromycin (Draxxin®; Zoetis Inc., New York, NY) subcutaneously in the thoracic region caudal to elbow (first injection at left elbow and second injection at right elbow) twice on Day 0 and Day 7 (7-day interval), immediately after the evening milking. Doses were calculated based on body weights collected the day before injection. Health status and injection-site reactions were monitored daily for 64 days after the first injection.

Individual doe milk samples were collected twice daily during regular milking times (morning milking at 6:30 a.m. and evening milking at 5:30 p.m.) for days 1–21 after the first injection, then once every 48 h at the morning milking for days 22–36 after the first injection, and every 72 h at the morning milking for days 37–64 after the first injection. Does were machine-milked; milk was collected using a commercial milk meter/sampler (Waikato Milking Systems, Hamilton, New Zealand) and the milk weights were recorded. Milk samples (~10–13 mL/sample) were transferred into 15 mL conical tubes, frozen at −20 °C within 30 min of collection, and then stored at −80 °C until analysis. Blood was collected twice daily, following each milking, during the first 7 days after the second injection via jugular venipuncture into heparinized blood tubes. Tubes were placed on ice, centrifuged at 1200 g for 5 min at 4 °C, and plasma samples were manually harvested and transferred to storage tubes, which were then immediately frozen at −20 °C and then stored at a −80 °C freezer until analysis. All animal procedures were approved in advance by the Animal Care and Use Committee of the University of California, Davis, CA.

### Tulathromycin analysis

Plasma tulathromycin concentrations were determined using the liquid chromatography mass spectrometry (LC-MS) method described in Galer et al. [13], while milk samples were analyzed for the concentrations of CP-60,300, a common fragment of tulathromycin and its metabolites, with an LC-MS method described by Boner et al. [14] because CP-60,300 is the marker residue of tulathromycin and all current US FDA guidelines on tulathromycin tolerances in the milk or target tissues are based on CP-60,300. Briefly, the LC was an Acquity® UPLC from Waters (Milford, MA) and the MS was a Thermo TSQ Quantum Discovery Max (Thermo Fisher Scientific, Waltham, MA) with a heated electrospray ionization source. Sample injections were prepared on an Ace C8, 2.1 × 50 mm, 3 μm column (MacMod, Chadds Ford, PA). Positive ions were monitored in the selected reaction monitoring (SRM) mode for CP-60,300 and an internal standard (a structurally similar compound provided by Pfizer [New York City, NY]) with transitions from 577.2 to 420.2 and from 591.3 to 434.2, respectively. For tulathromycin, the positive ions were monitored in the SRM mode with the doubly charged precursor to product ion pair of 404.1 to 578.0. The limit of detection (LOD) was 1.8 and 1.2 ng/mL for milk and plasma, respectively, and the limit of quantification (LOQ) was 5.0 ng/mL for milk and 4.0 ng/mL for plasma. The average inter-assay variation was measured by relative standard deviation (%RSD) and was 6.6% for milk and 11.3% for plasma. The milk quality control concentrations were 6, 60, and 600 ng/ml, and the plasma quality control concentrations were 15, 225, and 450 ng/ml. The recovery was 100% for milk and 96.7% for plasma.

### Pharmacokinetic analysis

Milk and plasma tulathromycin concentration-time data were analyzed for each individual animal using a non-compartmental approach (Phoenix WinNonlin®, version 6.4, Certara Inc., Cary, NC). Furthermore, tulathromycin plasma concentration and cumulative drug amount excreted in milk data were analyzed using the NLME-PK modeling approach in Phoenix NLME (version 1.3, Certara Inc., Cary, NC). The extended least-squares, first-order conditional estimation method (FOCE-ELS) with interaction was employed to fit the data to the model and calculate the pharmacokinetic parameters. Typical population pharmacokinetic parameter values, interindividual variability (IIV), residue error, and 95% confidence interval were calculated [15, 16]. Between-individual and between-occasion variability was assessed with an exponential model. Residual variability was tested using the additive, proportional, and combined error models. Compartment model selection was guided by goodness-of-fit plots (e.g., observed vs. predicted plasma concentrations and milk cumulative amounts, weighted residuals vs. predicted concentrations/amounts, and weighted residuals vs. time), the −2 log-likelihood (−2LL), Akaike information criterion (AIC), as well as the Bayesian information criterion (BIC). The model was selected on the basis of smaller values of AIC, better precision of estimates, and superior goodness-of-fit plots.

In order to determine whether the dosing regimen (single vs. twice injections) has an effect on tulathromycin depletion, plasma concentration and milk amount data of tulathromycin after a single SC injection in lactating goats (*n* = 8) from Grismer et al. [12] were included in the NLME-PK analysis. Briefly, eight adult (2–5 years) lactating dairy goats (body weight range: 72.5-89.0 kg) in their first to fourth lactation that met the inclusion criteria as described above were used in this study. Goats were injected with a single dose of 2.5 mg/kg tulathromycin (Draxxin®; Zoetis Inc., New York, NY) subcutaneously in the left thoracic region caudal to the elbow. Individual milk samples were collected starting at 12 h after injection twice daily during regular milking times (6 a.m. and 5 p.m.) from days 1–14, then once every 48 h (a.m. milking time) from days 14–28, and then every 72 h (a.m. milking time) from days 28–45 post injection. Blood samples were collected twice daily after each milking during the first 7 days after injection via jugular venipuncture into heparinized tubes. In addition, due to lack of absorption phase (<12 h) concentration data in the present study and the study by Grismer et al. [12], raw data from another study by Clothier et al. [3] where tulathromycin plasma concentrations were measured as early as 1 min after a single SC injection (2.5 mg/kg) in market age goats (*n* = 10), was also incorporated into the NLME-PK analysis. In brief, five daily mixed breed and five Boer male (intact and castrated; 6-month-old) goats received one SC injection of 2.5 mg/kg tulathromycin (Draxxin®; Pfizer Animal Health, New York, NY). Blood samples were collected from the jugular vein at 1, 2, 4, 6, 8, and 10 min, as well as at 0.25, 0.5, 1, 2, 4, 8, 12, 24, 48, 72, 96, 120, 144, 168, 192, 216, 240, 264, 288, 312, 336, and 360 h after injection. Plasma tulathromycin and milk CP-60,300 concentrations were analyzed using the same methods as described above.

### Simultaneous modeling of plasma and milk data

**Fig. 1:**
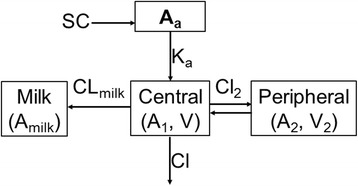
Schematic representation of the final pharmacokinetic model for plasma tulathromycin concentrations and milk cumulative excreted amounts of tulathromycin marker residue CP-60,300 in lactating goats. *A*_1_ is the total amount in the central compartment (ng); *A*_*a*_ is the amount in the absorption compartment via SC injection (ng); *A*_2_ is the total amount in peripheral compartment (ng); *A*_*milk*_ is the cumulative excreted amount in the milk (ng); *Cl* is central clearance (mL/h); *Cl*_2_ is clearance between the central and the peripheral compartment (mL/h); *Cl*_*milk*_ is clearance from the central compartment to the milk (mL/h); *K*_*a*_ is absorption rate constant after SC injection (1/h); *V* is volume of distribution for the central compartment (mL); *V*_2_ is volume of distribution for the peripheral compartment (mL)

The structural model for combined plasma and milk data was built sequentially. Firstly, a 1-, 2-, or 3-compartments model with first-order absorption and first-order elimination was fit to the plasma concentration data, and the best structural model was selected. Next, a milk compartment was added, and simultaneous modeling of plasma and milk data was then performed. The final pharmacokinetic structural model developed for tulathromycin plasma concentrations and milk cumulative amount excreted is shown in Fig. 1. Differential equations describing this pharmacokinetic model are shown below:

1 $$ \frac{d{A}_1}{dt}={A}_a\times {K}_a-\left(Cl\times C\right)-\left[Cl2\times \left(C-C2\right)\right]-\left(C{L}_{milk}\times C\right) $$

2 $$ \frac{d{A}_a}{dt}=-{A}_a\times {K}_a $$

3 $$ \frac{d{A}_2}{dt}=Cl2\times \left(C-C2\right) $$

4 $$ C={A}_1/V $$

5 $$ {C}_2={A}_2/{V}_2 $$

6 $$ \frac{d{A}_{milk}}{dt}=C{L}_{milk}\times C $$

where *A*_1_ is the total amount in the central compartment, *A*_*a*_ is the amount in the absorption compartment via SC injection, *A*_2_ is the total amount in peripheral compartment, *A*_*milk*_ is the cumulative amount excreted in the milk, *C* is the concentration in the central compartment, *C*_2_ is the concentration in the peripheral compartment. Definitions of pharmacokinetic parameters are provided in Table 3.

### Model evaluation

The performance and stability of the final pharmacokinetic model was evaluated with both graphical method by visually comparing predicted vs. observed data, as well as the bootstrap analysis. The bootstrap samples were collected through random sampling by replacing the original dataset to generate another dataset with the same sample size as the original but with a different combination of subjects. The bootstrap resampling analysis was repeated 100 times and conducted in Phoenix NLME. If the majority of the parameter estimates fell into the 95% confidence intervals (CIs) of the bootstrap values, the model was considered unbiased. However, if any parameter estimate was out of the 95% CI, possible reasons were explored and discussed.

### Model simulations

The final model was used in Phoenix NLME to conduct Monte Carlo simulations under dosing regimens of 2.5 mg/kg once or twice with 7-day interval via SC injection(s). For each dosing schedule, the Monte Carlo simulation generated time-concentration (or amount) profiles of tulathromycin in plasma and milk for 100 replicates. The simulated data were used to estimate the times when the upper limits of the 95% confidence intervals of simulated plasma and milk concentrations were below the tolerances. Simulated milk concentrations were calculated by dividing the simulated excreted amounts in milk by the measured milk volumes produced over the period of time between samples. There are no official tolerances for tulathromycin in lactating goats in the US or Europe, so the limits of detection (LODs) in plasma (1.2 ng/ml) and milk (1.8 ng/ml) were used as the most conservative endpoint.

## Results

### General health and pharmacokinetic data

All goats were healthy throughout the study with normal appetite and milk production levels. The body weight did not change significantly before nor at the end of the study. Four animals exhibited drug administration reactions, including vocalization, extension/flexion of the front limb, and/or looking back at the elbow. Similar to what we observed in our previous single injection study [12], injection-site reactions ranged from mild swelling to multiple skin lesions. All swellings diminished from their maximum size by the end of the study.

**Fig. 2:**
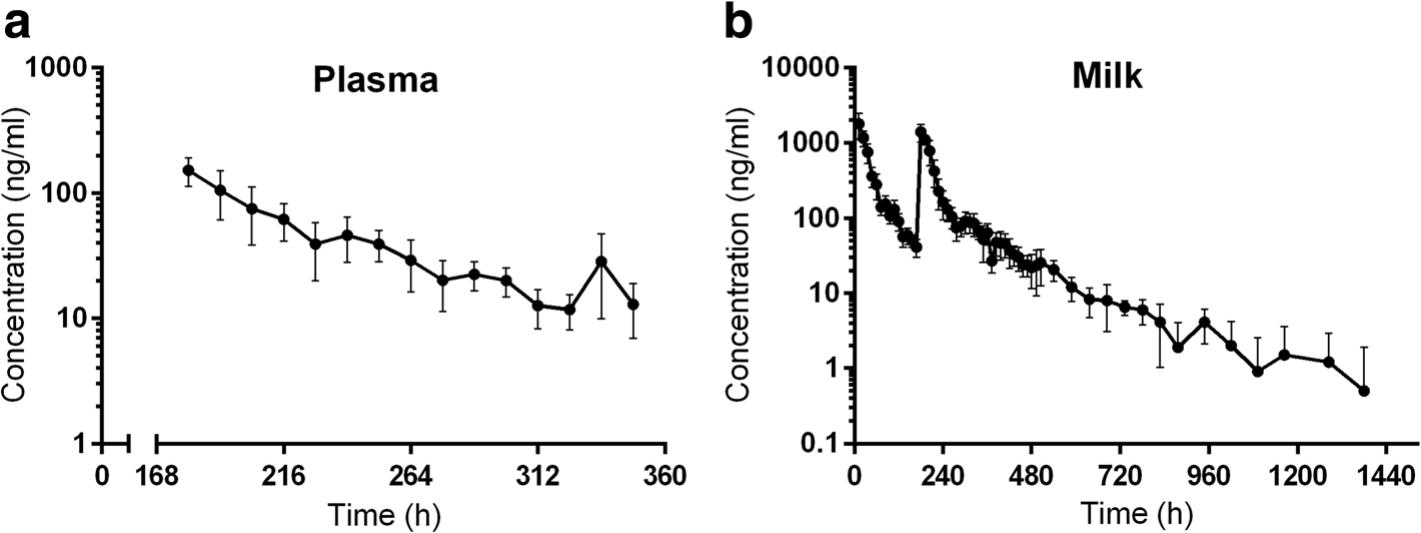
Plots of tulathromycin concentration versus time in plasma (**a**) and CP-60,300 concentration versus time in milk (**b**)

The plasma and milk concentration-time data collected during the current study are shown in Fig. 2. The average plasma and milk non-compartmental pharmacokinetic parameter values are shown in Table 1, while Table 2 shows the average plasma and milk concentration data. The plasma pharmacokinetic profile of tulathromycin after the second injection reported in this study were somewhat different numerically from that observed in the previous study using a different population of lactating goats (*n* = 8) after a single injection [12]. However, the pharmacokinetic parameter values between the first and the second injections could not be compared statistically because the data were from two different studies using two different populations of animals. The milk concentration and pharmacokinetic parameters were analyzed after both the first and the second injections in the present study. The elimination rate constant (λz) was lower and the elimination half-life (T_1/2λz_) longer after the second compared to the first injection; other parameters were not statistically different (Table 1).

**Table 1:** Noncompartmental pharmacokinetic parameters for tulathromycin in plasma and the marker residue CP-60,300 in milk of lactating goats following two subcutaneous injections of 2.5 mg/kg tulathromycin at 7-day interval

Pharmacokinetic parameters (units)	Plasma (injection 2 only)		Milk (injection 1)		Milk (injection 2)		
	mean	SD	mean	SD	mean	SD	*p* value^#^
C_max (obs)_ (ng/mL)	155.211	39.501	1806.280	660.824	1457.238	282.876	0.191
T_max (obs)_ (h)	16.828	8.880	15.016	5.240	17.930	9.017	0.574
λz (1/h)	0.013	0.002	0.017	0.006	0.005	0.001	<0.001
T_1/2λz_ (h)	56.421	8.265	51.752	35.768	158.002	40.291	<0.001
AUC_last_ (h*ng/mL)	8050.601	1532.931	61805.331	6351.608	68156.845	12472.633	0.220
AUC_inf_ (h*ng/mL)	9120.588	1599.754	65147.987	7889.685	68978.306	12598.494	0.478

C_max (obs)_: observed maximum plasma or milk concentration; T_max (obs)_: time to observed maximum plasma or milk concentration after each injection; λz: elimination rate constant; T_1/2λz_: elimination half-life; AUC_last_: area under the plasma or milk concentration vs. time curve to the last sampled value; AUC_inf_: area under the plasma or milk concentration vs. time curve extrapolated to infinity

^#^ *p* values of Student’s *t* test of the difference in the milk pharmacokinetic parameters between the first injection and the second injection

**Table 2:** Average concentrations of tulathromycin in plasma and the marker residue CP-60,300 in milk after twice subcutaneous injections of 2.5 mg/kg tulathromycin in lactating goats

Time	Plasma	SD	Milk	SD	Time	Plasma	SD	Milk	SD
12	NA	NA	1791.4	672.02	360	NA	NA	64.3	20.39
24	NA	NA	1167.0	264.23	372	NA	NA	26.9	8.23
36	NA	NA	763.7	226.64	384	NA	NA	47.7	16.54
48	NA	NA	364.8	109.83	396	NA	NA	46.7	18.93
60	NA	NA	281.1	103.50	408	NA	NA	45.9	16.43
72	NA	NA	140.3	30.71	420	NA	NA	37.0	15.74
84	NA	NA	154.1	45.25	432	NA	NA	32.8	10.11
96	NA	NA	107.1	23.03	444	NA	NA	30.3	10.55
108	NA	NA	131.4	39.94	456	NA	NA	24.0	7.51
120	NA	NA	90.6	23.20	468	NA	NA	23.9	7.56
132	NA	NA	56.7	15.54	480	NA	NA	22.1	10.49
144	NA	NA	59.2	14.50	492	NA	NA	23.3	14.07
156	NA	NA	50.2	9.61	504	NA	NA	25.4	12.87
168	NA	NA	41.0	11.11	540	NA	NA	20.6	6.28
180	152.5	39.03	1392.8	363.32	588	NA	NA	12.0	4.25
192	105.8	44.82	1096.9	151.09	636	NA	NA	8.3	3.55
204	75.1	36.48	789.3	292.69	684	NA	NA	8.0	4.91
216	61.9	20.64	424.9	168.10	732	NA	NA	6.5	1.42
228	39.3	19.24	230.1	101.49	780	NA	NA	6.0	2.21
240	46.2	18.20	164.0	68.47	828	NA	NA	4.1	3.06
252	39.3	10.90	131.2	41.73	876	NA	NA	1.9	2.12
264	29.1	12.87	105.3	33.04	948	NA	NA	4.1	1.98
276	20.1	8.77	74.7	24.81	1020	NA	NA	2.0	2.21
288	22.5	5.94	78.3	21.99	1092	NA	NA	0.9	1.66
300	20.0	5.20	91.6	29.83	1164	NA	NA	1.5	2.11
312	12.6	4.34	88.9	26.16	1284	NA	NA	1.2	1.71
324	11.7	3.66	85.2	28.94	1380	NA	NA	0.5	1.40
336	28.5	18.61	68.4	16.72	1452	NA	NA	0.0	0.00
348	12.9	5.94	52.5	26.43	1524	NA	NA	0.0	0.00

*Time* time after the first injection (h), *Plasma* average concentration of tulathromycin in the plasma (ng/mL), *Milk* average concentration of CP-60,300 in the milk (ng/mL), *SD* standard deviation, *NA* not applicable as no sample was taken

### Structural NLME-PK model development

Results of model comparison and evaluation between the 3-compartment and 2-compartment models based on the data in lactating goats only are provided in Additional file 1: Figure S1. In addition, comparisons of model evaluation results of the initial 2-compartment model (without the absorption phase data) and the final 2-compartment model (with the absorption phase data from [3]) are shown in Additional file 2: Figure S2. Overall, a 2-compartment model with first-order absorption and first-order elimination best characterized the plasma concentration data. A total of 232 plasma concentrations of tulathromycin in lactating goats and 300 plasma concentrations in market meat goats were simultaneously modeled. The plasma pharmacokinetic model parameters are provided in Table 3. The distribution of residual variability was most adequately characterized with a proportional error model. A total of 411 milk cumulative excreted amounts were added to develop the milk pharmacokinetic model.

**Table 3:** Pharmacokinetic model parameters for plasma tulathromycin and milk CP-60,300 concentrations in lactating goats

Parameter	Description	Population mean	IIV	Bootstrap value	2.5% CI	97.5% CI
V (mL)	Volume of distribution for the central compartment	326627	1.072	260011.65	169213.19	369548.19
Cl (mL/h)	Central clearance	21109.2	0.184	19214.724	16224.881	22463.998
V2 (mL)	Volume of distribution for the peripheral compartment	676758	0.232	721665.92	567400.38	835445.28
Cl2 (mL/h)	Clearance between the central and the peripheral compartment	12550.5	0.710	16958.522	11499.469	24340.243
CLmilk (mL/h)	Clearance from the central compartment to the milk	882.461	0.121	816.285	686.264	957.191
Ka (1/h)	Absorption rate constant after subcutaneous (SC) injection	0.295	6.255	0.938	0.327	2.318
Residue error 1 (%)	Proportional error model of tulathromycin concentrations in plasma	0.485	NA	0.484	0.419	0.549
Residue error 2 (%)	Proportional error model of CP-60,300 cumulative amounts in milk	0.0234	NA	0.0228	0.0157	0.0279

*CI* confidence interval, *IIV* interindividual variability, *NA* not available

### Model evaluation

**Fig. 3:**
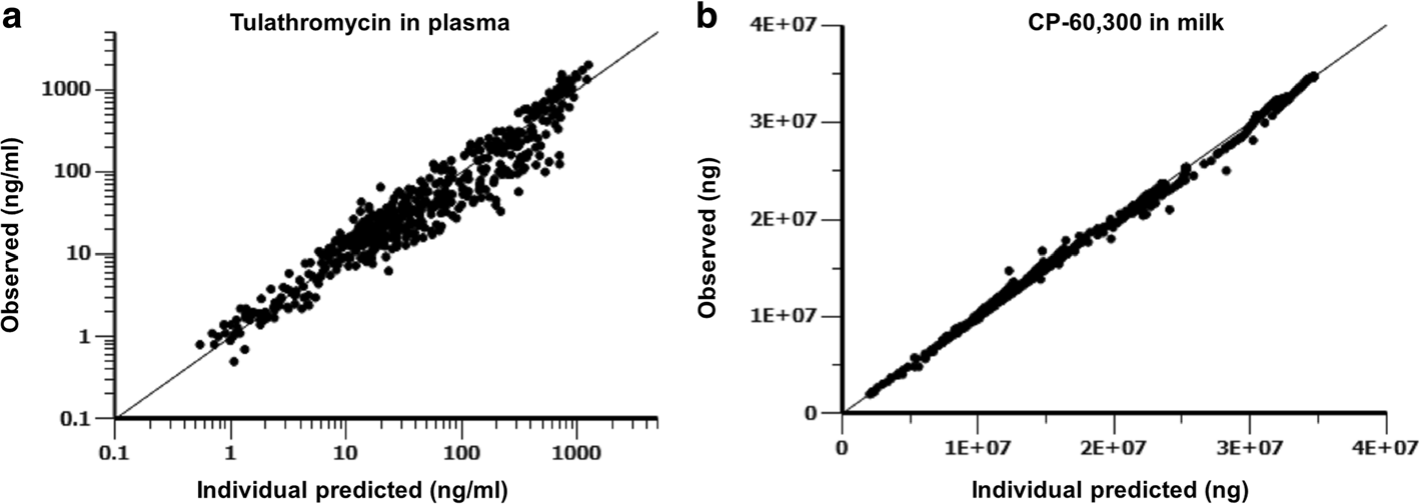
Goodness-of-fit plots of plasma tulathromycin concentrations and milk cumulative excreted amounts of tulathromycin marker residue CP-60,300 in lactating goats. Comparisons between observed versus individual predicted tulathromycin concentrations or CP-60,300 amounts in the plasma (**a**) and milk (**b**), respectively

**Fig. 4:**
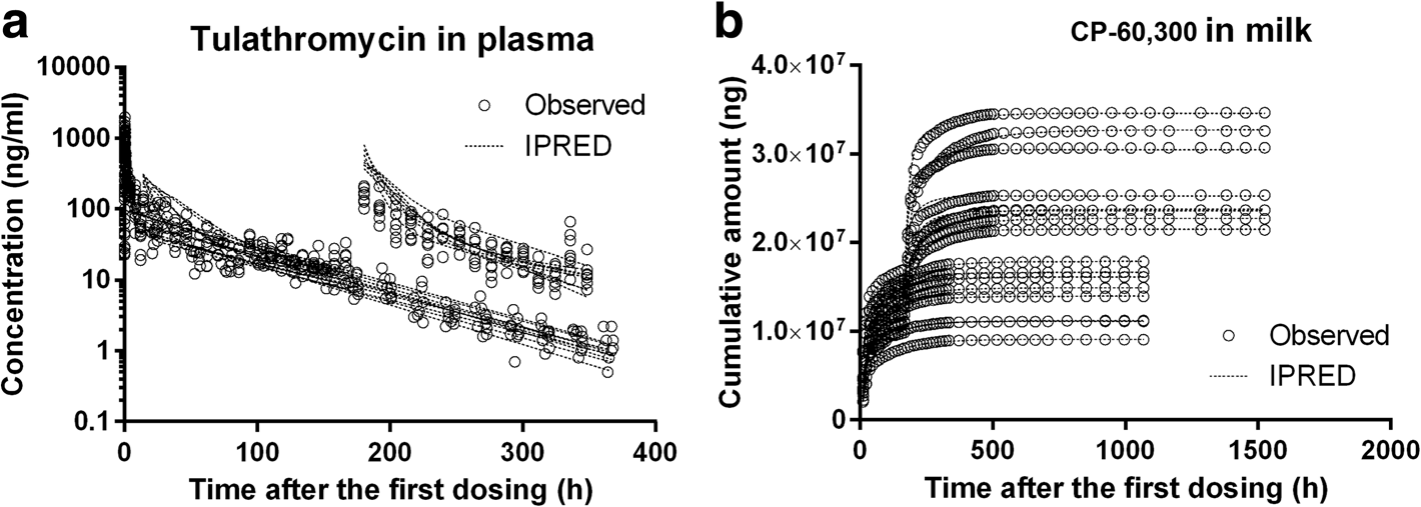
Comparisons of observed versus individual predicted time-concentration profiles in plasma (**a**) and time-cumulative excreted amount profiles in milk (**b**) of lactating goats. Unfilled circles represent experimentally observed data from Clothier et al. [3], Grismer et al. [12], and the present pharmacokinetic study. Dotted lines represent model simulation results

Goodness-of-fit plots from the final pharmacokinetic model are presented in Fig. 3. The plots displayed an excellent correlation between the model-predicted and observed values. Figure 4 shows the comparisons of time-concentration (or amount) profiles vs. the individual predicted data in plasma and milk. The model adequately captured the pharmacokinetic profiles of tulathromycin and CP-60,300 in plasma and milk, respectively.

### Model simulations

**Fig. 5:**
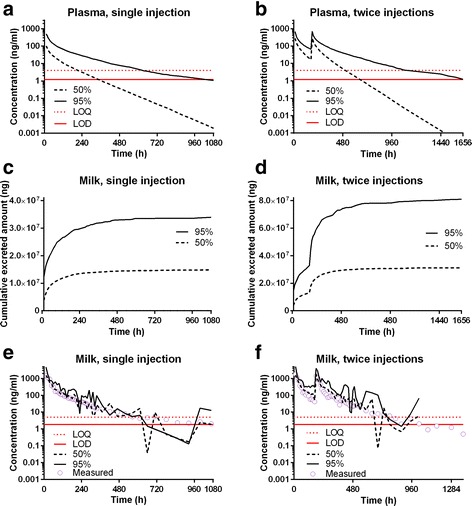
Simulated data for the plasma tulathromycin concentrations (**a**, **b**), cumulative excreted amounts of marker residue CP-60,300 in milk (**c**, **d**), milk concentrations (**e**, **f**) of CP-60,300 in lactating goats after single or twice injections. The *solid and dashed black lines* represent the 95th and 50th percentiles of the simulated data, respectively. The *solid and dotted red lines* represent limit of detection (LOD) and limit of quantification (LOQ), respectively, in milk (LOD = 1.8 ng/mL, LOQ = 5 ng/ml) and plasma (LOD = 1.2 ng/mL, LOQ = 4 ng/ml). The *empty purple circles* represent the measured data from Grismer et al. [12] and the present pharmacokinetic study

The simulated time-concentration (or amount) profiles of tulathromycin in plasma and the marker residue CP-60,300 in milk from the final pharmacokinetic model are shown in Fig. 5. Based on the time-concentration profiles and LOD in plasma, it was estimated with a 95% confidence that the plasma concentration was below LOD 43 days after a single SC injection, and 62 days after the second injection in a 2-injection regimen (7-day interval) (Fig. 5a, b).

The simulated time-cumulative amounts of CP-60,300 in milk displayed a hyperbolic increase at the early phase after injection, started to reach a plateau on ~25 days after the first injection, and continuously increased slowly even up to the last simulation time points, which were day 64 after the single injection (Fig. 5c, d). The simulated time-concentration profiles in milk fluctuated throughout the simulation period (Fig. 5e, f). However, the overall trend of decreasing in the concentrations of CP-60,300 in milk after injection was still observed and the simulated concentrations matched well with the measured concentrations. The fluctuating milk concentrations at the terminal kinetic phase prevented from estimating milk withdrawal time for tulathromycin in goats. Therefore, a definite conclusion on the milk withdrawal time recommendation cannot be made from this study. Nevertheless, based on the experimental data of CP-60,300 in milk, concentrations of the marker residue above the LOD were not measured in the milk beyond 45 days after the single injection [12], and 39 days after the second injection in the 2-injection regimen (7-day interval) for the present studied population of animals.

## Discussion

This study reports plasma and milk pharmacokinetic data up to 2 months after twice SC injections of 2.5 mg/kg tulathromycin with a 7-day interval in lactating goats. There were palpable injection-site reactions, but no substantial adverse clinical signs occurred; the body weight and milk production were not affected negatively. These observations suggest that twice SC injections of 2.5 mg/kg tulathromycin do not cause clinical adverse reactions in lactating goats, and could be considered for treating lung infections in lactating goats provided that extra-label drug use requirements are met.

Based on the new data in this study and previously published single SC injection pharmacokinetic data in goats [3, 12], a NLME-PK model for tulathromycin in lactating goats was created. The model-predicted plasma concentration and milk cumulative excreted amount data correlated well with the experimental data (Figs. 3 and 4). The model is an excellent tool to estimate tulathromycin plasma and milk depletion profiles (Fig. 5). However, one limitation is that the model can only predict the cumulative excreted amounts in milk and the milk concentrations of tulathromycin marker residue have to be calculated by dividing the measured volumes of milk samples, which were different between different milking periods (range 7–14 lbs/day). This was one of the reasons why the model-predicted milk concentrations of tulathromycin marker residue exhibited a fluctuating pattern at the terminal phase, which was consistent with the measured data (Table 2).

The model predicted that a very long period (62 days) would be needed to ensure that plasma concentrations of tulathromycin and milk concentrations of the marker residue fell below the LOD in 95% of the population following two administrations of tulathromycin 7 days apart. One of the reasons for this is that the model was built using time-concentration data from several studies with variable characteristics such as age, body weight, and milk production. Unfortunately, data for these factors were not reported for all the studies, so explanatory co-variates for the pharmacokinetic variability could not be included in the final model. The pharmacokinetic parameters’ variability therefore represents the variability across all the included studies and, as a result, the model predicted a wide range of concentrations to encompass the full 95% of the study population.

Note that the milk concentration analysis was based on the marker residue CP-60,300, which is a common fragment of both the parent compound and metabolites of tulathromycin. In contrast, only the parent tulathromycin was measured in the plasma. This is a limitation of the current study, since it is based on the assumption that the ratio between parent drug concentrations in the plasma and total residues in the milk is constant. However, this approach to the analysis may be a contributing factor to the fluctuating milk concentrations that were observed in the study (i.e., the milk concentration may represent mainly the parent drug at the early kinetic phase and then primarily the metabolites at the later phase). To further optimize the present model, additional studies using more sensitive and specific analytical method are needed to determine milk concentrations of both tulathromycin and the marker residue CP-60,300. In addition, high milk:plasma partition coefficient (~10) and relatively high protein bound percentage (~50%) of tulathromycin in goats may contribute to the fluctuating milk concentrations at the terminal phase [10, 12].

As introduced before, another helpful approach in the estimation of withdrawal times is PBPK modeling, which can be used to predict both the concentrations and the cumulative excreted amounts of drugs in the milk [17, 18]. Therefore, future studies that extend the published tulathromycin PBPK model in juvenile and market-age meat goats [10] to lactating goats are needed to compare the estimated withdrawal intervals from different approaches (NLME-PK vs. PBPK), and then to determine a more accurate estimate to protect food safety.

The estimated times when plasma tulathromycin concentrations were below 1.2 ng/mL LOD were 43 days after a single SC injection and 62 days after the second injection in the 2-injection regimen. This estimation was based on the LOD because the tolerance for tulathromycin in goat milk is not available in the US or Europe. Tulathromycin marker residue LOD in milk is much lower than the tolerance of 5.5 ppm (5500 ng/ml) in cattle liver (the target tissue). However, cattle liver tolerance is not appropriate to be used to estimate milk withdrawal time because milk is consumed on a regular basis and in large quantities compared to cattle liver. Consequently, a conservative estimate based on goat milk-specific data is necessary in order to provide a precautionary withdrawal time estimation.

Our previous study reported that a withdrawal interval of at least 45 days are needed when tulathromycin is administered to lactating goats SC at a dose of 2.5 mg/kg [12]. However, plasma/milk sampling did not extend beyond 45 days after injection, nor was an NLME-PK model developed to estimate the upper limit of the 95% confidence intervals of plasma and milk concentrations. The present study confirms the previous study finding and further demonstrate that for twice SC administrations of tulathromycin a withdrawal interval of 39 days after the second injection (i.e., 46 days after the first injection) is needed for milk concentrations to fall below tolerance (i.e., LOD if official tolerance is not available) for the studied population of animals. Additionally, our NLME-PK model simulations showed that 62 days after the second injection in the 2-injection regimen are needed for plasma concentration to fall below the tolerance for 95% of the simulated population. It should be noted that the longer estimated time for plasma to fall below tolerance than the time for milk was because the former has taken into account the population variability, whereas the later was simply based on the present experiment using a limited number of animals. Other laboratories have reported lower LODs (0.46-0.7 ng/mL in plasma) using more sensitive liquid chromatography tandem mass spectrometry (LC-MS/MS) methods [8, 9, 19]. Based on LOD of 0.7 ng/mL in plasma, the estimated time when plasma concentrations fall below LOD will be >70 days after the second injection in the 2-injection paradigm.

## Conclusions

In summary, this study suggests that, although two SC injections of 2.5 mg/kg tulathromycin is a safe treatment for pulmonary infections in lactating goats, there is considerable potential for contamination of the human food supply unless a prolonged and extended withdrawal interval is observed. The experimental data showed that the marker residue CP-60,300 concentrations were below LOD in all milk samples from the studied animals at 39 days after the second injection. However, a much longer withdrawal time of 62 days would be needed to ensure tulathromycin concentrations in both plasma and milk fall below the LOD in 95% of the population. These estimated times are much longer than the current meat withdrawal time recommendation of 18 days in non-lactating cattle and of 5 days for swine. Therefore, tulathromycin use in lactating goats should be cautious and an extended withdrawal period should be considered. Nevertheless, a definite milk withdrawal time recommendation for a large diverse population of lactating goats cannot be made due to the fluctuating milk concentration at the terminal kinetic phase and milk samples should be tested before milk is allowed to enter the human food chain. Additional studies using more sensitive and specific analytical method to determine milk concentrations of both tulathromycin and the marker residue CP-60,300 and using Bayesian population PBPK modeling approaches are needed to provide a comparative, and potentially, more conservative milk withdrawal time estimation.

## Additional files

Additional file 1: Figure S1. Model comparison initial 3- vs. initial 2-compartment model. (PPTX 224 kb)
Additional file 2: Figure S2. Model comparison initial 2- vs. final 2-compartment model. (PPTX 258 kb)
Additional file 3: All plasma tulathromycin and milk CP-60,300 concentration data used in the model simulation. (XLSX 84 kb)

## Abbreviations

CI: Confidence interval
IM: Intramuscular
IV: Intravenous
LC-MS: Liquid chromatography mass spectrometry
LOD: Limit of detection
LOQ: Limit of quantification
MIC: Minimal inhibitory concentration
NLME-PK: Nonlinear mixed-effects pharmacokinetic
PBPK: Physiologically based pharmacokinetic
SC: Subcutaneous
SRM: Selected reaction monitoring
