# Supplementary material for: Estimation of tulathromycin depletion in plasma and milk after subcutaneous injection in lactating goats using a nonlinear mixed-effects pharmacokinetic modeling approach
Source: BMC Vet Res. 2016 Nov 18;12:258. doi: 10.1186/s12917-016-0884-4 (PMC5116175; doi:10.1186/s12917-016-0884-4)
Supplement: Additional file 1: Figure S1. — Model comparison initial 3- vs. initial 2-compartment model. (PPTX 224 kb) [file 12917_2016_884_MOESM1_ESM.pptx]

## Slide 1
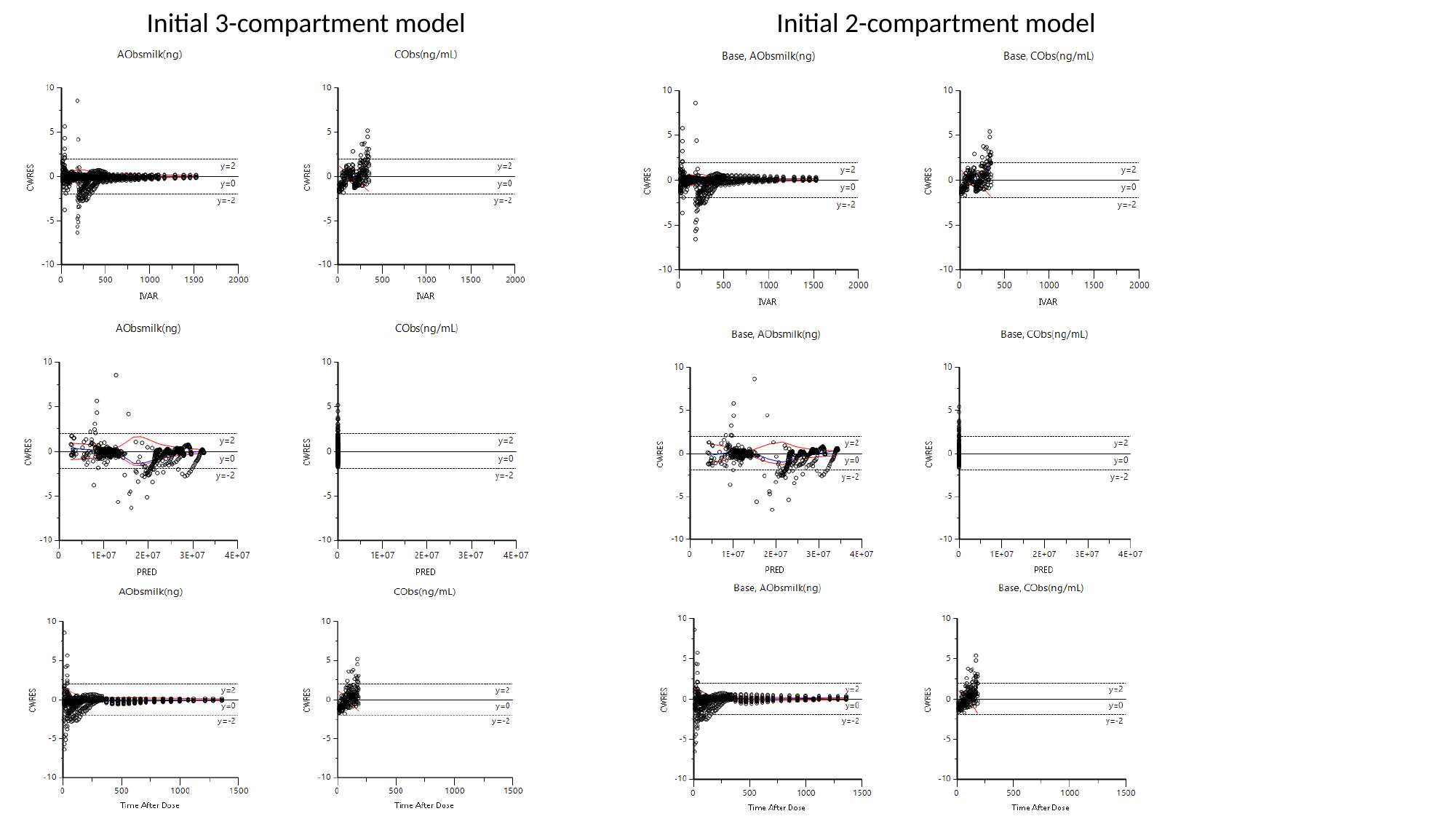

Initial 3-compartment model
Initial 2-compartment model

## Slide 2
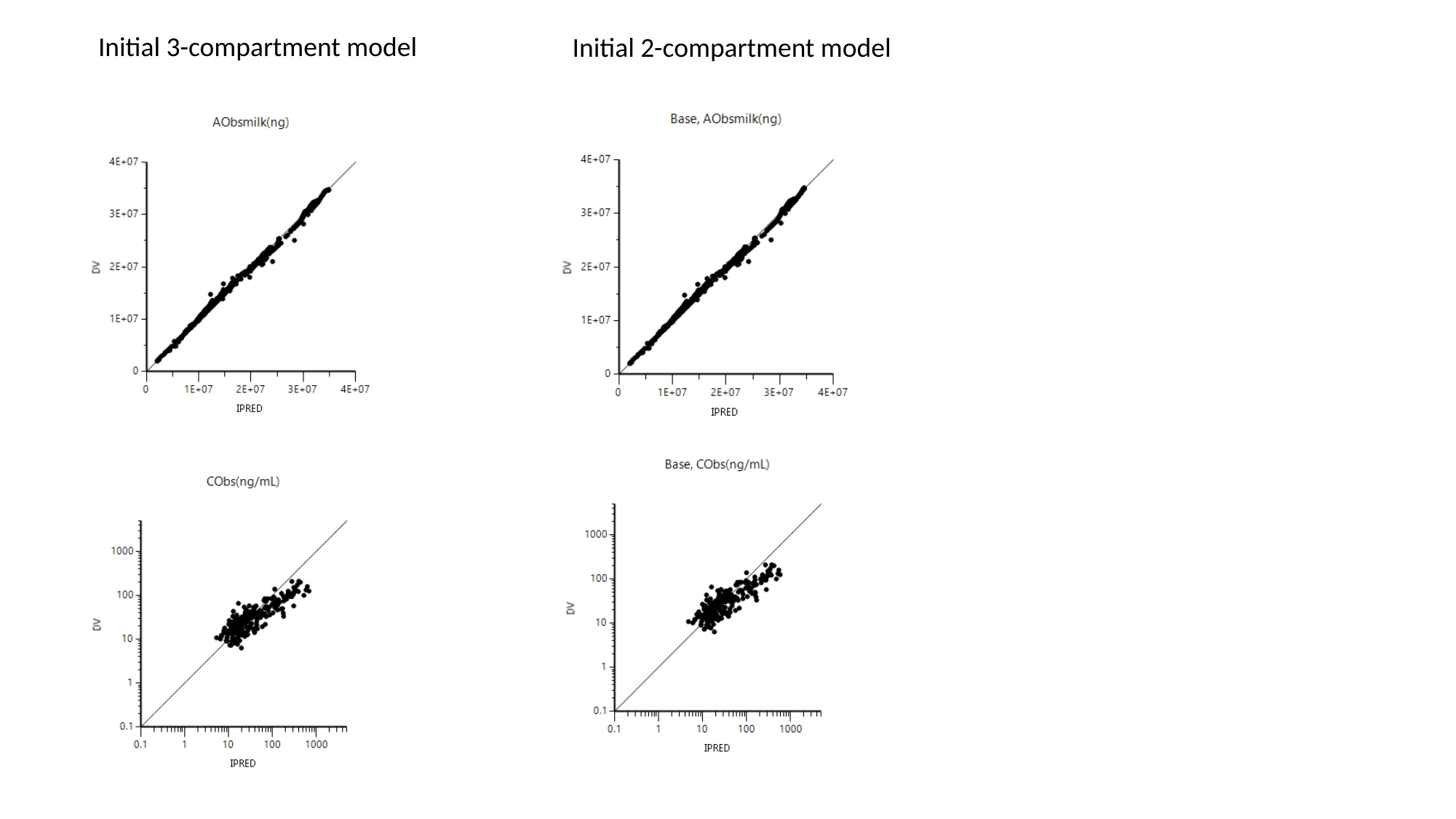

Initial 3-compartment model
Initial 2-compartment model

## Slide 3
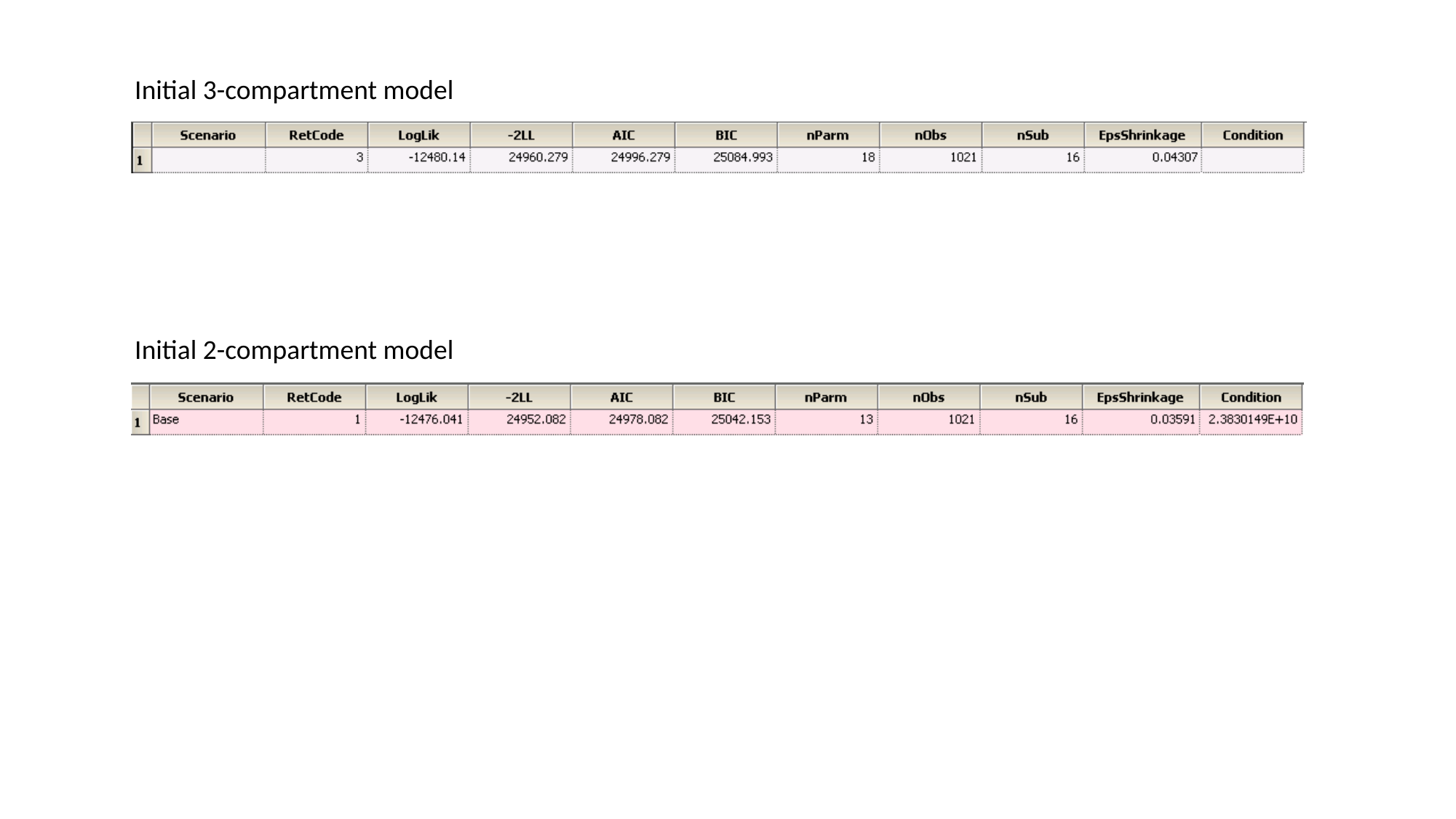

Initial 3-compartment model
Initial 2-compartment model
