# Supplementary material for: Estimation of tulathromycin depletion in plasma and milk after subcutaneous injection in lactating goats using a nonlinear mixed-effects pharmacokinetic modeling approach
Source: BMC Vet Res. 2016 Nov 18;12:258. doi: 10.1186/s12917-016-0884-4 (PMC5116175; doi:10.1186/s12917-016-0884-4)
Supplement: Additional file 2: Figure S2. — Model comparison initial 2- vs. final 2-compartment model. (PPTX 258 kb) [file 12917_2016_884_MOESM2_ESM.pptx]

## Slide 1
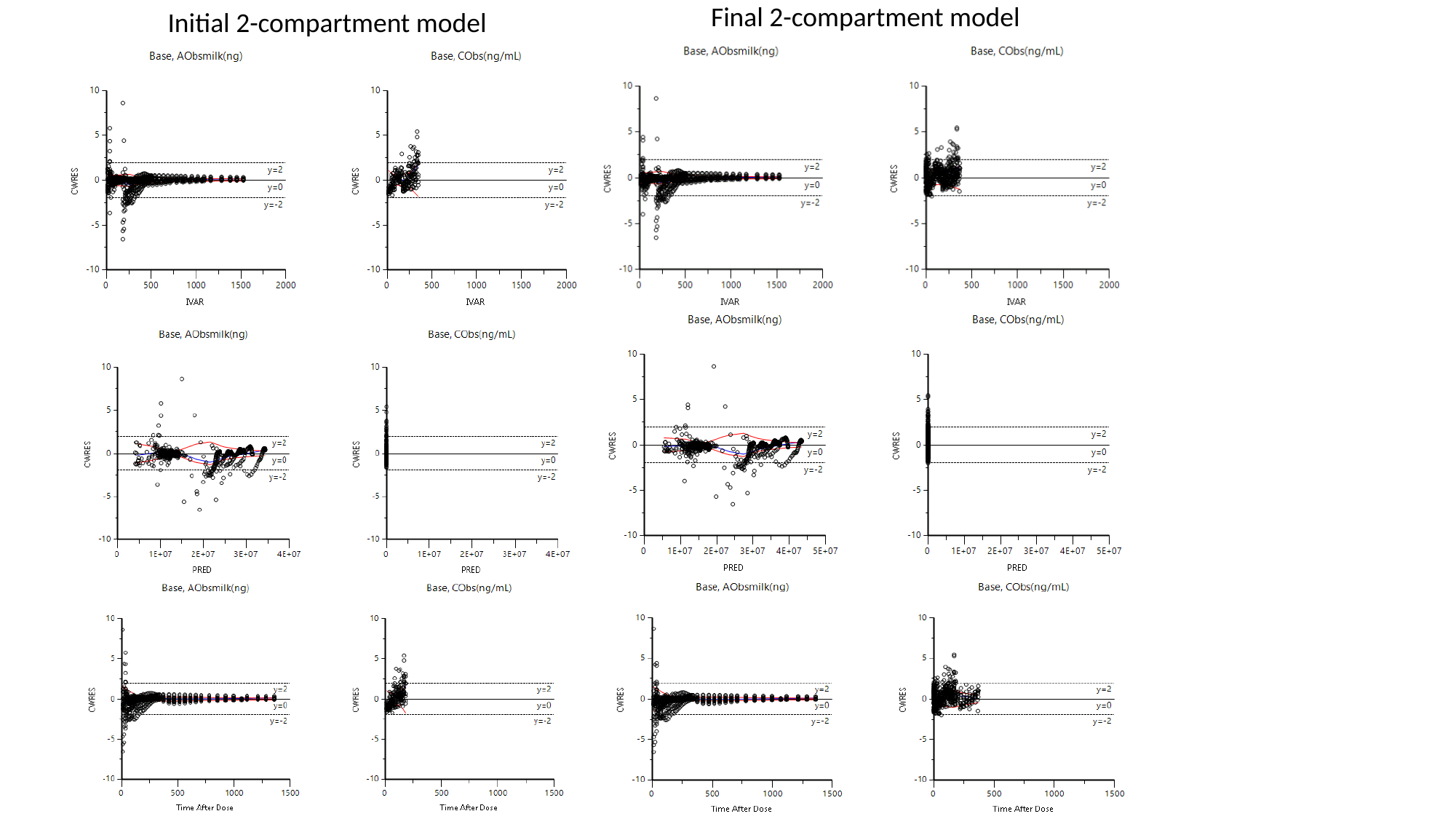

Initial 2-compartment model
Final 2-compartment model

## Slide 2
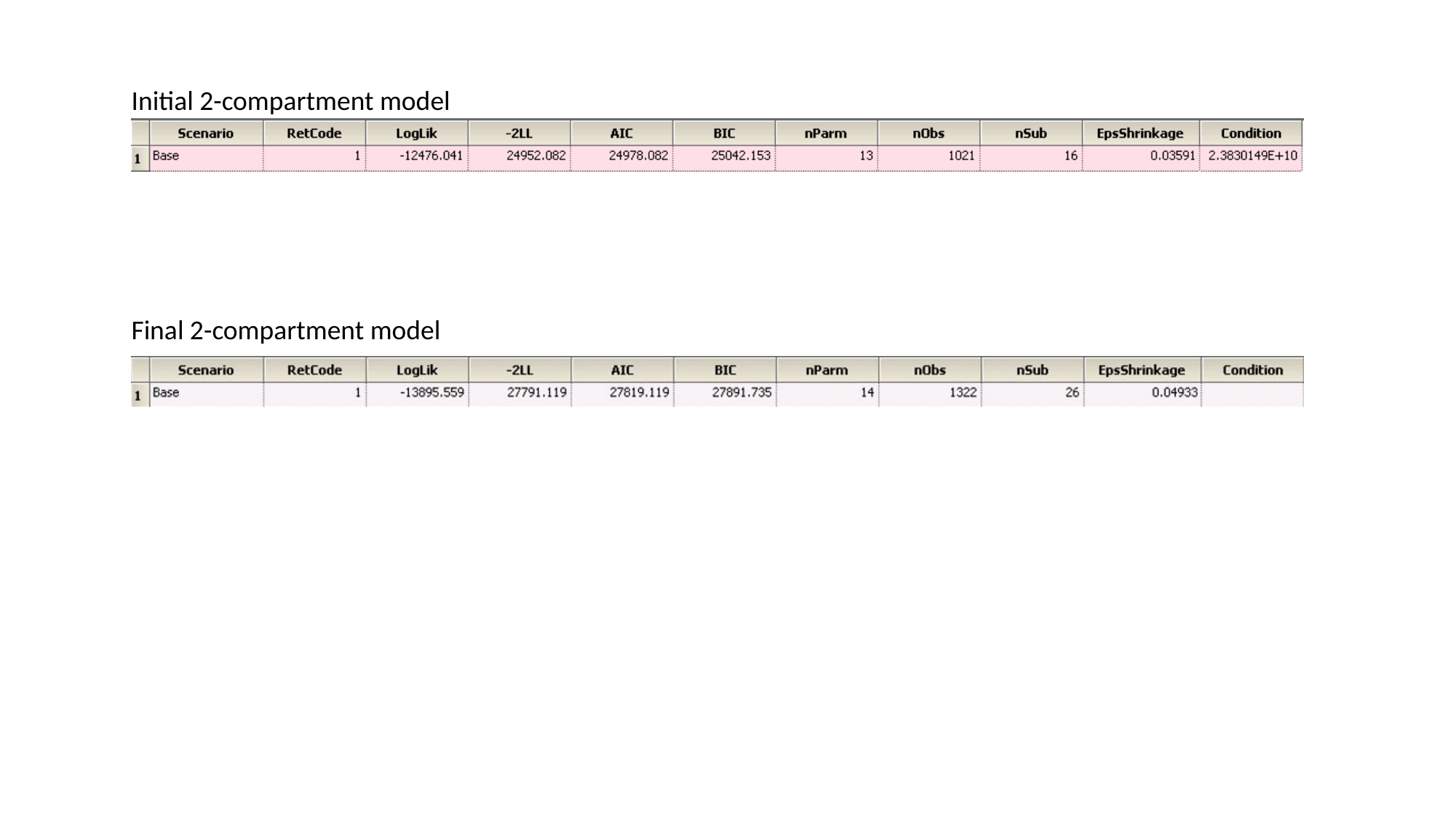

Initial 2-compartment model
Final 2-compartment model

## Slide 3
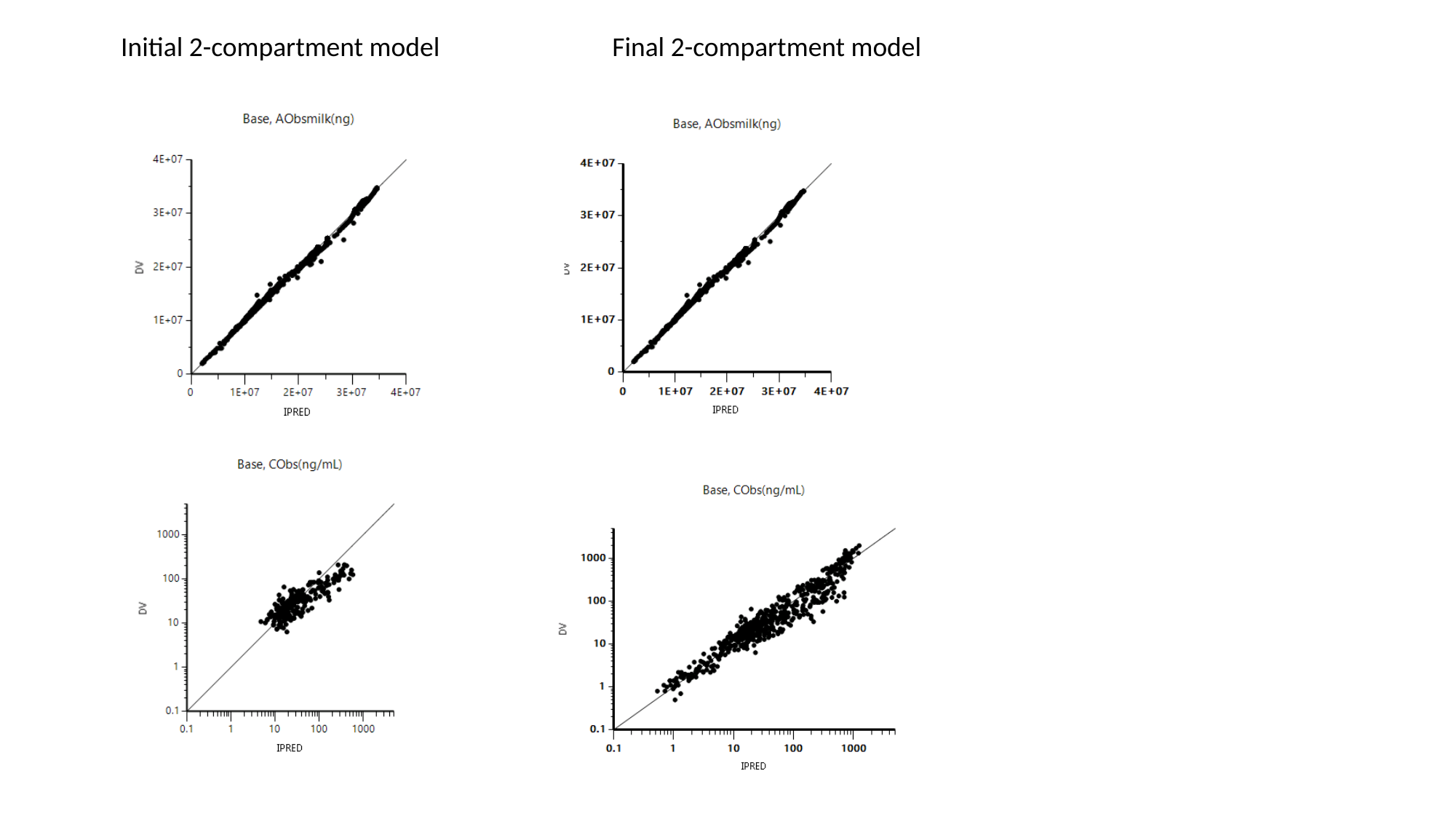

Initial 2-compartment model
Final 2-compartment model
